# Addressing Information Biases Within Electronic Health Record Data to Improve the Examination of Epidemiologic Associations With Diabetes Prevalence Among Young Adults: Cross-Sectional Study

**DOI:** 10.2196/58085

**Published:** 2024-10-01

**Authors:** Sarah Conderino, Rebecca Anthopolos, Sandra S Albrecht, Shannon M Farley, Jasmin Divers, Andrea R Titus, Lorna E Thorpe

**Affiliations:** 1 Department of Population Health, New York University Grossman School of Medicine, New York, NY, United States; 2 Department of Epidemiology, Mailman School of Public Health at Columbia University, New York, NY, United States; 3 ICAP at Columbia University, New York, NY, United States; 4 Department of Foundations of Medicine, New York University Long Island School of Medicine, Mineola, NY, United States

**Keywords:** information bias, electronic health record, EHR, epidemiologic method, confounding factor, diabetes, epidemiology, young adult, cross-sectional study, risk factor, asthma, race, ethnicity, diabetic, diabetic adult

## Abstract

**Background:**

Electronic health records (EHRs) are increasingly used for epidemiologic research to advance public health practice. However, key variables are susceptible to missing data or misclassification within EHRs, including demographic information or disease status, which could affect the estimation of disease prevalence or risk factor associations.

**Objective:**

In this paper, we applied methods from the literature on missing data and causal inference to assess whether we could mitigate information biases when estimating measures of association between potential risk factors and diabetes among a patient population of New York City young adults.

**Methods:**

We estimated the odds ratio (OR) for diabetes by race or ethnicity and asthma status using EHR data from NYU Langone Health. Methods from the missing data and causal inference literature were then applied to assess the ability to control for misclassification of health outcomes in the EHR data. We compared EHR-based associations with associations observed from 2 national health surveys, the Behavioral Risk Factor Surveillance System (BRFSS) and the National Health and Nutrition Examination Survey, representing traditional public health surveillance systems.

**Results:**

Observed EHR-based associations between race or ethnicity and diabetes were comparable to health survey-based estimates, but the association between asthma and diabetes was significantly overestimated (OR_EHR_ 3.01, 95% CI 2.86-3.18 vs OR_BRFSS_ 1.23, 95% CI 1.09-1.40). Missing data and causal inference methods reduced information biases in these estimates, yielding relative differences from traditional estimates below 50% (OR_MissingData_ 1.79, 95% CI 1.67-1.92 and OR_Causal_ 1.42, 95% CI 1.34-1.51).

**Conclusions:**

Findings suggest that without bias adjustment, EHR analyses may yield biased measures of association, driven in part by subgroup differences in health care use. However, applying missing data or causal inference frameworks can help control for and, importantly, characterize residual information biases in these estimates.

## Introduction

Understanding patterns and risk factors of chronic disease burden is a key function of public health practice. Electronic health record (EHR) data have numerous strengths that can be leveraged for this purpose, including near real-time information on large patient populations, allowing for improved precision and timeliness when estimating patterns or trends in disease burden [1]. EHRs also contain clinically based diagnoses, laboratory results, and physical measurements [2]. Often, researchers use these clinical data to classify patients’ disease status using rule-based computable phenotypes or prespecified logic-based criteria [3]. Using EHR-defined measures of disease offers promise for improving the estimation of patterns or associations compared to traditional surveillance systems (eg, health surveys), several of which have poor validity from self-reported disease status and limited reliability for small subgroups or geographies [4].

Despite these strengths, the classification of disease status using EHR data is susceptible to information biases. Misclassification or measurement errors in key variables can bias estimates of epidemiologic associations. Within EHR data, a diagnostic suspicion bias could occur if certain patients are disproportionately screened for health outcomes compared to others (eg, those who are obese may be more likely to be screened for diabetes with A_1c_ testing) [5], and an informed presence bias could occur if, for example, patients who visit the health system more frequently are sicker or have more opportunities to receive a diagnosis than infrequent visitors [6]. These different biases can distort our understanding of patterns or risk factors for disease burden, such as by underestimating the relative burden among individuals with limited or more fragmented access to care. Notably, selection biases whereby the EHR sample is not generalizable to the target population can also affect estimates of epidemiologic associations, and misclassification itself can be framed as a selection bias issue [7]. Improving our capacity to address misclassification, framed as an information bias issue herein, is a critical step to providing valid inferences.

A variety of statistical approaches and frameworks have been developed to help address misclassification, including treating misclassification as a missing data problem, a causal inference problem, or both [8,9]. With EHR-based computable phenotypes, researchers often rely on variables that are not identifiably missing (eg, lack of diagnosis codes are assumed to mean the absence of disease). For example, pertinent evidence of the disease may be absent from a single EHR due to patients receiving care across multiple distinct health care systems, which could lead to individuals being falsely classified as disease-free [5]. Internal or external data sets can allow for the validation and correction of the computable phenotype’s performance, but these data sets are often costly or resource-intensive to obtain [8,10]. Under a missing data framework, the observed health outcome can be assumed to have some level of misclassification and the true health outcome for some or all of the patients can be treated as missing [8,11]. Traditional missing data methods, such as regression calibration, multiple imputation, and inverse probability weighting (IPW), can then be used to address this misclassification [8,10-12].

Misclassification in disease status can also be conceptualized using directed acyclic graphs (DAGs), illustrating factors that affect the causal relationship between the true and observed exposure and outcome [8,13]. Researchers can use traditional epidemiologic methods to control for variables that act as confounders of the observed exposure and outcome. For example, researchers often hypothesize that the number of health care encounters affects the misclassification of EHR-defined disease status through an informed presence bias, with EHR samples further restricted to those with at least 1 encounter [6]. Prior studies have demonstrated that conditioning on the number of encounters can sometimes reduce confounding from differential misclassification but also has the potential to induce a smaller Berkson’s or M-bias if the number of encounters is a common effect of the exposure and outcome, particularly when computable phenotypes are highly sensitive [14]. Nevertheless, DAGs offer a promising approach to address misclassification.

In this paper, we applied missing data and causal inference frameworks to evaluate the impact of information biases in EHR data on longstanding epidemiologic research questions about diabetes among young adults. Diabetes is a serious, chronic condition that is still relatively rare within this age group, affecting an estimated 3% of those aged 18‐44 years [15] but is increasing in both prevalence and incidence [16-18]. We first aimed to estimate the age and sex-adjusted odds of diabetes by race or ethnicity, given established differences in diabetes prevalence across racial or ethnic groups [15,19,20]. Second, we characterized the association between asthma and diabetes, presuming a causal relationship between these chronic conditions [21]. For the target population of in-care US adults aged 18‐44 years, we compared estimated associations using these frameworks to estimates based on probability samples from national health surveys. This work informs the broader discussion on how to address differential misclassification of disease outcomes within EHR data.

## Methods

### EHR Sample

The EHR sample comprises patients from NYU Langone Health, a large academic medical center with primary service areas in the New York City boroughs of Manhattan, Brooklyn, and Queens. EHR data were pulled from the Epic Clarity database for all New York City–resident patients aged 18‐44 years who had an inpatient or outpatient encounter from 2017 to 2019.

Demographic variables of age, sex (male or female), race or ethnicity (White, Black, Latino, Asian, and other), most recent insurance status (Medicaid vs non-Medicaid), and neighborhood poverty level (<10%, 10%-<20%, 20%-<30%, and ≥30% living in poverty within resident census tract) were defined for each patient. Race or ethnicity was imputed for those with unknown race or ethnicity (19.4%, n=88,102) using the Bayesian Improved Surname Geocoding (BISG) methods through the “wru” R package (R Core Team) [22]. Neighborhood poverty level was assigned using zip code tabulation area poverty group when census tract level data were unavailable (1%). Those with an unknown or other age or sex (<1%) were excluded from all analyses.

Health care use variables of total encounters, duration within the NYU Langone Health system, presence of at least 1 routine health exam (2024 ICD-10-CM: Z00.*), presence of at least 1 diabetes-related laboratory (fasting glucose, random glucose, or A_1c_), and presence of at least 1 encounter with an endocrinology review of systems, or inventory of signs or symptoms of diseases related to the endocrine system, were also defined. Endocrinology review of systems were identified using keyword text searches of history and physical examination notes and progress notes (Multimedia Appendix 1).

Patients were classified as having prevalent obesity, asthma, and diabetes if they had evidence supporting these chronic conditions, using all historical EHR data through 2019, and were classified as not having each respective health outcome without such evidence. In alignment with diabetes definitions from national health surveys, EHR-defined diabetes included all diabetes types. Evidence of diabetes included at least 2 encounter diagnoses for diabetes (ICD-10-CM: E08.*, E09.*, E10.*, E11.*, and E13.*); or 1 encounter diagnosis and at least 2 elevated A_1c_ laboratory results ≥6.5%; or at least 1 antidiabetes prescription medication (not including metformin or acarbose) [23]. Evidence of asthma was defined as at least 2 encounter diagnoses for asthma (ICD-10-CM: J45*–J46*) or at least 2 prescriptions for asthma-related medications [24]. To maintain consistency across chronic disease classification methods, evidence of obesity was defined as a most recent BMI ≥30 kg/m^2^, with no naïve corrections for those who were missing BMI, height, or weight measurements (19.1%, n=86,709).

### EHR-Based Estimation

We estimated odds ratios (OR) for diabetes by race or ethnicity and asthma status under 4 EHR-based estimation methods that we describe herein. First, “naïve” models were estimated by fitting a logistic regression model for observed diabetes status (DM*) on the total sample (n=454,612). ORs for race or ethnicity were adjusted for age and sex and ORs for asthma were adjusted for the potential confounders of age, sex, race or ethnicity, Medicaid insurance status, obesity, and neighborhood poverty level, as informed by existing literature [25-27].

Second, “sufficiency” models were estimated among the subset of patients who we hypothesized to have sufficient data, defined as those with at least 1 encounter with an endocrinology review of systems or those who were classified as diabetic through the above definition (n=181,036). Since diabetes is a rare disease within the young adult population, we assumed that the specificity of the classification was near-perfect and all patients who were classified as diabetic had sufficient data [28]. Sensitivity analyses tested this assumption and varied the definition of sufficient data to incorporate information related to the other health outcomes (eg, having a diabetes-related laboratory, BMI measurement, and respiratory review of systems; Multimedia Appendix 1). Sufficiency models were estimated by fitting a logistic regression model for DM* using the same covariates as the naïve models.

Third, using “IPW” models, we hypothesized that missing health outcomes would be predicted by demographics (eg, differential screening by race or ethnicity), health care use (eg, informed presence bias), and neighborhood (eg, degree of continuity of care within the health system by catchment area). We estimated the probability of having sufficient data using a multilevel logistic regression model including all demographic and health care use variables and a random intercept for neighborhood defined by public use microdata areas. Stabilized IPW weights were then calculated as the inverse of the predicted probability of having sufficient data multiplied by the overall probability of having sufficient data [10]. The final models were estimated by fitting a logistic regression model for DM* on the subset of patients defined as having sufficient data (n=181,036), weighted for the stabilized IPW weights and using the same covariates as the naïve models.

Fourth, using DAG models (Multimedia Appendix 1), we hypothesized that total encounters would both be associated with differential misclassification of health outcomes through an informed presence bias and be a common effect of the health outcomes, consistent with prior research [6,14]. DAG models were estimated by fitting a logistic regression model for DM* using the total sample (n=454,612), controlling for total encounters and the covariates included in the naïve model. Further details on these models can be found in Multimedia Appendix 1.

### Comparison to Survey-Based Estimation

For comparison to traditional surveillance systems, samples were obtained from 2 publicly available national health surveys, the 2019 Behavioral Risk Factor Surveillance System (BRFSS) and the pooled 2013-March 2020 National Health and Nutrition Examination Survey (NHANES). BRFSS is a cross-sectional telephone survey conducted by the Centers for Disease Control and Prevention annually on a sample of over 400,000 US adults [29]. Within BRFSS data, diabetes and asthma were defined by self-reported prior diagnosis from a medical provider, and obesity was defined by a BMI ≥30 kg/m^2^ based on self-reported height and weight. Demographic variables of 5-year age group, sex (male or female), race or ethnicity (White, Black, Latino, Asian, and other), insurance status (uninsured vs insured), and income level (<US $50,000, US $50,000-<US $75,000, ≥US $75,000) were defined for each respondent. To reduce the effects of undiagnosed diabetes on misclassification of self-reported diabetes status, the BRFSS survey data were subset to those aged 18‐44 years who were in care, as defined as those who reported having a personal health care provider.

NHANES is a cross-sectional survey involving interviews and physical examinations that is conducted by the Centers for Disease Control and Prevention annually on a sample of approximately 5000 US children and adults [30]. Within NHANES data, diabetes was defined by a self-reported prior diagnosis or elevated laboratory results (A_1c_≥6.5% or fasting glucose≥126 mm Hg) [15,31]. Sensitivity analyses varied this definition to be based solely on self-reported prior diagnosis. Asthma was defined by self-reported prior diagnosis from a medical provider and obesity was defined by a measured BMI≥30 kg/m^2^. Demographic variables of age, sex (male or female), race or ethnicity (White, Black, Latino, Asian, and other), insurance status (Medicaid vs non-Medicaid), and income level (≤130%, 130%‐350%, >350% of the federal poverty level) were defined for each respondent. NHANES data were subset to those aged 18‐44 years.

Survey-based estimates were obtained by fitting logistic regression models for diabetes accounting for the unequal probability sample, stratification, and clustering in the complex sample designs [32,33]. Survey-based ORs for race or ethnicity were adjusted for age and sex and ORs for asthma were adjusted for age, sex, race or ethnicity, obesity, insurance status, and income level. Relative differences between EHR-based and survey-based ORs were calculated as percent differences.

### Ethical Considerations

This study was approved by the NYU Langone Health Institutional Review Board (i20-01338) and Columbia University Institutional Review Board (AAAU5390) and informed consent was waived. Participants were not compensated.

## Results

### EHR Sample

The EHR sample comprised 454,612 patients seen within the NYU Langone Health system from 2017 to 2019 (Table 1). A total of 37.8% (n=171,968) of patients were male and 22.2% (n=100,979) had Medicaid insurance. The largest racial or ethnic group within the sample was White, with 41.8% (n=190,225) having a White race or ethnicity recorded within the EHR, and 52.1% (n=237,057) classified as White through BISG imputation. Approximately one-quarter (n=115,249) of patients had a routine medical exam and one-half (n=205,408) had a DM-related laboratory. Within the full sample, 3.1% (n=14,044) of patients were classified as having diabetes, 17.5% (n=79,580) were classified as being obese, and 4.2% (n=19,240) were classified as having asthma.

**Table 1.** Descriptive summary of the New York University patient population by data sufficiency status^a^^,^^b^.

Variables	Total sample (N=454,612)	Insufficient case (n=273,576)	Sufficient case^a^ (n=181,036)
Age (years), mean (SD)	32.13 (7.11)	32.02 (7.13)	32.31 (7.07)
Sex (male), n (%)	171,968 (37.8)	100,964 (36.9)	71,004 (39.2)
Medicaid insurance, n (%)	100,979 (22.2)	59,001 (21.6)	41,978 (23.2)
**Raw race or ethnicity, n (%)**			
White	190,225 (41.8)	111,123 (40.6)	79,102 (43.7)
Black	45,509 (10.0)	24,442 (8.9)	21,067 (11.6)
Latino	62,989 (13.9)	32,157 (11.8)	30,832 (17.0)
Asian or Pacific Islander	35,262 (7.8)	20,947 (7.7)	14,315 (7.9)
Other	32,525 (7.2)	19,669 (7.2)	12,856 (7.1)
Missing	88,102 (19.4)	65,238 (23.8)	22,864 (12.6)
**Imputed race or ethnicity, n (%)**			
White	237,057 (52.1)	144,783 (52.9)	92,274 (51.0)
Black	57,709 (12.7)	33,439 (12.2)	24,270 (13.4)
Latino	86,679 (19.1)	49,131 (18.0)	37,548 (20.7)
Asian or Pacific Islander	49,170 (10.8)	31,288 (11.4)	17,882 (9.9)
Other	23,997 (5.3)	14,935 (5.5)	9062 (5.0)
Recorded BMI, n (%)	367,903 (80.9)	190,916 (69.8)	176,987 (97.8)
Encounters^c^, mean (SD)	15.23 (23.51)	9.41 (13.15)	24.03 (31.60)
Duration^d^, mean (SD)	1.84 (1.93)	1.53 (1.83)	2.31 (1.98)
Routine medical exam, n (%)	115,249 (25.4)	32,786 (12.0)	82,463 (45.6)
Diabetes-related laboratory^b^, n (%)	205,408 (45.2)	80,728 (29.5)	124,680 (68.9)
**Neighborhood coverage** ^**e**^ **, n (%)**			
<10%	79,563 (17.5)	48,320 (17.7)	31,243 (17.3)
10%-<20%	143,907 (31.7)	82,148 (30.0)	61,759 (34.1)
20%-<30%	163,076 (35.9)	101,293 (37.0)	61,783 (34.1)
30%-<40%	68,066 (15.0)	41,815 (15.3)	26,251 (14.5)
Asthma, n (%)	19,240 (4.2)	6167 (2.3)	13,073 (7.2)
Obese, n (%)	79,580 (17.5)	38,819 (14.2)	40,761 (22.5)

a Sufficient cases defined as those with at least 1 encounter with an endocrinology review of systems or those who were classified as diabetic through the computable phenotype of having at least 2 encounter diagnoses for diabetes, 1 encounter diagnosis and at least 2 elevated A_1c_ laboratory results ≥6.5%, or at least 1 antidiabetes prescription medication.

b Including all A_1c_, random blood glucose, and fasting blood glucose laboratory results*.*

c Number of encounters.

d Number of years in the health system.

e Proportion of individuals residing in the Public Use Microdata Area (PUMA) neighborhood who are present within the electronic health record system.

A total of 39.8% (n=181,036) of the patient population were classified as having sufficient data (Table 1). Patients who were classified as sufficient had greater health care use, measured by a higher average number of total encounters, greater duration within the NYU system, and greater proportion having at least 1 BMI, routine health exam, or diabetes-related laboratory. Compared to insufficient cases, a greater proportion also had a known race or ethnicity (87.4%, n=158,172) or were classified as diabetic (7.8%, n=14,044), obese (22.5%, n=40,761), or asthmatic (7.2%, n=13,073).

Within the total naïve EHR sample, those who were classified as nondiabetic consistently had lower health care use than those who were classified as diabetic (Table 2). This pattern was disrupted in the sufficiency sample, where a greater proportion of patients who are nondiabetic had at least 1 routine medical exam (46.7%, n=77,927 vs 32.3%, n=4536 of patients who are diabetic ) and almost all patients had a recorded BMI regardless of diabetes status. Within both the naïve and sufficiency samples, a lower proportion of those classified as nondiabetic were of Black or Latino race or ethnicity and were classified as having asthma or obesity compared to those classified as diabetic.

**Table 2.** Descriptive summary of the New York University patient population by diabetes status^a^^,^ ^b^ .

Variables	Naïve—diabetes status		Sufficient—diabetes status^a^	
	Nondiabetic (n=440,568)	Diabetic (n=14,044)	Nondiabetic (n=166,992)	Diabetic (n=14,044)
Age (30‐44 years), n (%)	273,216 (62.0)	10,843 (77.2)	104,005 (62.3)	10,843 (77.2)
Sex (male), n (%)	166,204 (37.7)	5764 (41.0)	65,240 (39.1)	5764 (41.0)
Medicaid insurance, n (%)	96,760 (22.0)	4219 (30.0)	37,759 (22.6)	4219 (30.0)
**Raw race or ethnicity, n (%)**				
White	185,119 (42.0)	5106 (36.4)	73,996 (44.3)	5106 (36.4)
Black	43,289 (9.8)	2220 (15.8)	18,847 (11.3)	2220 (15.8)
Latino	59,636 (13.5)	3353 (23.9)	27,479 (16.5)	3353 (23.9)
Asian or Pacific Islander	34,174 (7.8)	1088 (7.7)	13,227 (7.9)	1088 (7.7)
Other	31,378 (7.1)	1147 (8.2)	11,709 (7.0)	1147 (8.2)
Missing	86,972 (19.7)	1130 (8.0)	21,734 (13.0)	1130 (8.0)
**Imputed race or ethnicity, n (%)**				
White	231,412 (52.5)	5645 (40.2)	86,629 (51.9)	5645 (40.2)
Black	55,268 (12.5)	2441 (17.4)	21,829 (13.1)	2441 (17.4)
Latino	82,827 (18.8)	3852 (27.4)	33,696 (20.2)	3852 (27.4)
Asian or Pacific Islander	47,887 (10.9)	1283 (9.1)	16,599 (9.9)	1283 (9.1)
Other	23,174 (5.3)	823 (5.9)	8239 (4.9)	823 (5.9)
Recorded BMI, n (%)	354,043 (80.4)	13,860 (98.7)	163,127 (97.7)	13,860 (98.7)
Obese, n (%)	73,412 (16.7)	6168 (43.9)	34,593 (20.7)	6168 (43.9)
Encounters^c^, mean (SD)	14.29 (21.16)	44.90 (54.27)	22.27 (28.19)	44.90 (54.27)
Duration^d^, mean (SD)	14.29 (21.16)	2.87 (2.06)	2.26 (1.97)	2.87 (2.06)
Routine medical exam, n (%)	110,713 (25.1)	4536 (32.3)	77,927 (46.7)	4536 (32.3)
Diabetes-related laboratory^b^, n (%)	193,480 (43.9)	11,928 (84.9)	112,752 (67.5)	11,928 (84.9)
**Neighborhood coverage** ^**e**^ **, n (%)**				
<10%	76,463 (17.4)	3100 (22.1)	28,143 (16.9)	3100 (22.1)
10%-<20%	139,333 (31.6)	4574 (32.6)	57,185 (34.2)	4574 (32.6)
20%-<30%	158,890 (36.1)	4186 (29.8)	57,597 (34.5)	4186 (29.8)
30%-<40%	65,882 (15.0)	2184 (15.6)	24,067 (14.4)	2184 (15.6)
Asthma, n (%)	17,339 (3.9)	1901 (13.5)	11,172 (6.7)	1901 (13.5)

a Sufficient cases defined as those with at least 1 encounter with an endocrinology review of systems or those who were classified as diabetic through the computable phenotype of having at least 2 encounter diagnoses for diabetes, 1 encounter diagnosis and at least 2 elevated A_1c_ laboratory results ≥6.5%, or at least 1 antidiabetes prescription medication.

b Including all A_1c_, random blood glucose, and fasting blood glucose laboratory results.

c Number of encounters.

d Number of years in the health system.

e Proportion of individuals residing in the Public Use Microdata Area (PUMA) neighborhood who are present within the electronic health record system.

### Estimated Associations With Race or Ethnicity

**Figure 1.**
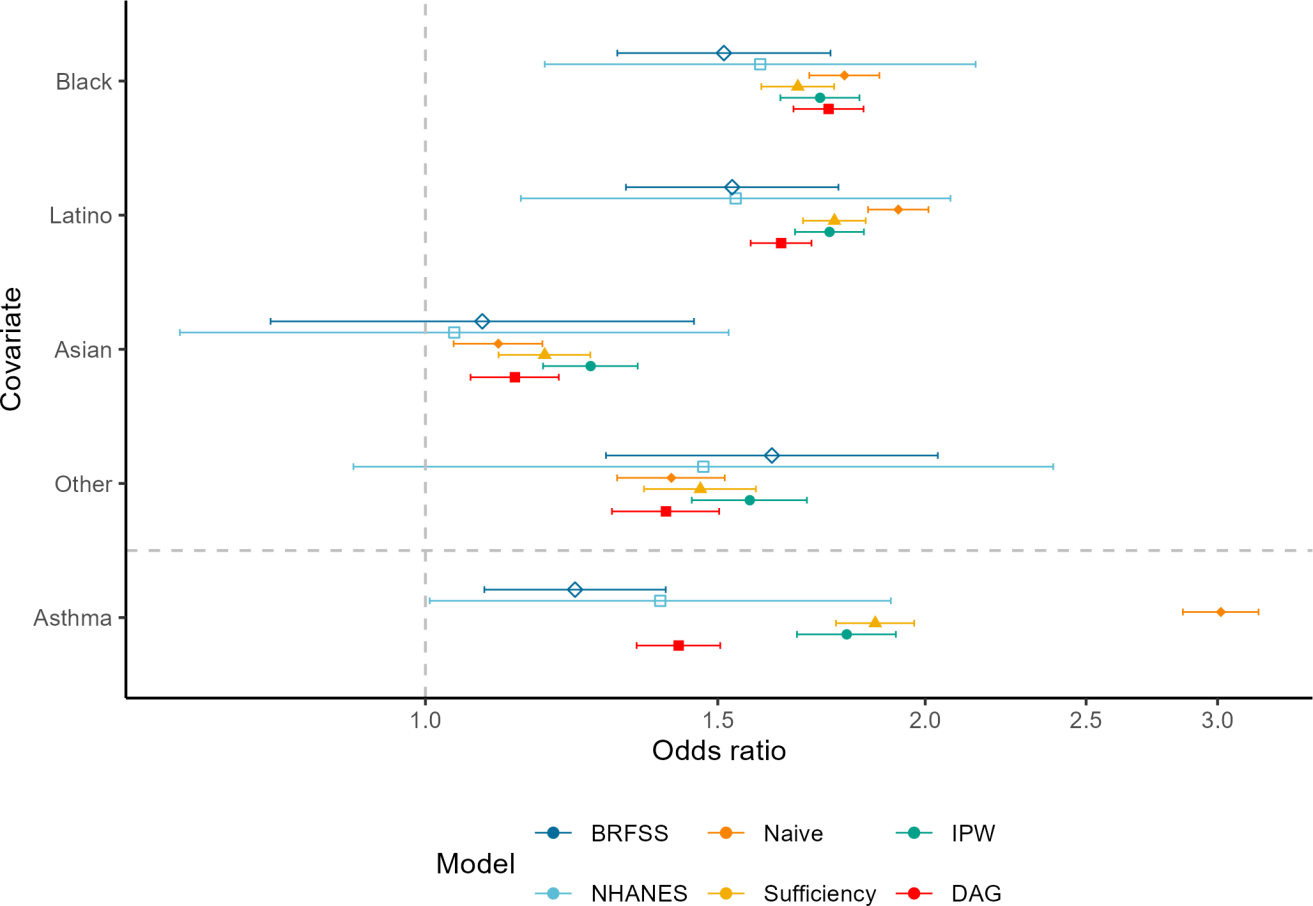
Odds ratios for diabetes by race or ethnicity and asthma, EHR-based estimates versus health survey estimates. BRFSS: Behavioral Risk Factor Surveillance System; DAG: directed acyclic graph; EHR: electronic health record; IPW: inverse probability weighting; NHANES: National Health and Nutrition Examination Survey.

Estimated associations between race or ethnicity and diabetes were comparable across BRFSS and NHANES surveys (Figure 1). In both the survey and EHR-based analyses, respondents who were Black or Latino had significantly higher odds of diabetes compared to White respondents, controlling for age and sex. The naive EHR-based OR estimate for Latino patients was 26% higher than the BRFSS point estimate (OR_Naive_ 1.93, 95% CI 1.85‐2.01 vs OR_BRFSS_ 1.53, 95% CI 1.32‐1.77). Sufficiency, IPW, and causal methods reduced this association, with point estimates falling within the 95% CIs and relative differences below 15% compared to both health survey estimates. BRFSS and NHANES respondents who were Asian did not have significantly higher odds of diabetes compared to White respondents, and CIs were wide due to the small sample sizes of this subgroup. Within the EHR analyses, patients who were Asian had a significant 11%‐26% increased odds of diabetes.

### Estimated Associations With Asthma

In the BRFSS and NHANES analyses, having asthma was associated with an approximate 20%‐40% increased odds of diabetes after controlling for demographics and obesity (OR_BRFSS_ 1.23, 95% CI 1.09‐1.40 and OR_NHANES_ 1.38, 95% CI 1.01‐1.91). In the naive EHR analysis, asthma was strongly associated with diabetes, with those with asthma estimated to have 3 times the odds of diabetes as those without asthma after controlling for demographics and obesity (95% CI 2.86‐3.18). This association was reduced in the sufficiency sample and the IPW-weighted sample, with corrected OR estimates falling within the 95% CI of the NHANES estimate and having an approximate 30%‐50% relative difference from the health survey point estimates. The association between asthma and diabetes was further reduced in the DAG model (OR 1.42, 95% CI 1.34‐1.51), falling within the 95% CIs of both survey-based estimates and having a 3% relative difference from the NHANES point estimate and a 15% relative difference from the BRFSS point estimate (Figure 1). Sensitivity analyses varying the definition for sufficiency (Table S3 in Multimedia Appendix 1), varying the BRFSS inclusion criteria, or varying the NHANES diabetes definition produced similar patterns in these results (Table S1 in Multimedia Appendix 1).

## Discussion

### Principal Findings

Our analysis explored the potential impact of information biases on observed associations of diabetes risk factors within an EHR sample of young adults. We estimated naïve associations on the full patient sample and then attempted to address information biases using missing data and causal inference frameworks. Biases were apparent in the naïve association between asthma and diabetes, which was significantly higher than the health survey-based estimates used as a benchmark for the expected association. All EHR-based methods resulted in estimated associations between race or ethnicity and diabetes that were largely comparable to health survey-based estimates. Those who were observed to have diabetes had greater health care use than those who were classified as nondiabetic, which could reflect an information bias where those with a greater number of health care encounters may have been more likely to have documentation of an underlying diabetes or asthma diagnosis. Attempting to address this bias through missing data or causal frameworks reduced the estimated associations between asthma and diabetes, with the causal framework having the best performance in producing an estimate comparable to the benchmarks.

Within naïve analyses, the observed age and sex-adjusted ORs for diabetes among Latino patients appeared slightly inflated compared to health survey estimates. Using IPW, or controlling the number of health care encounters produced ORs that were closer to health survey estimates. Despite the potential to introduce collider bias, subsetting to those with sufficient data also led to estimated associations that were more similar to health survey estimates than the naïve method. Prior research has demonstrated that Latino and Black individuals may have increased screening for diabetes while Asian individuals may have decreased screening compared to White individuals [34]. These disparities in screening practices may partially explain the observed patterns within the EHR estimates. Increased likelihood of screening would produce a positive bias while decreased likelihood of screening would produce a negative bias in naïve EHR associations. The tested methods may have helped correct for this bias by controlling or restricting based on factors associated with the likelihood of diabetes screening, resulting in decreases in the Latino ORs and increases in the Asian OR relative to naïve estimates.

Consistent with prior research, the naïve association between 2 EHR-observed conditions, asthma and diabetes, was substantially positively biased relative to health survey estimates and prior studies from the literature [5,6,14,21]. Imposing data sufficiency criteria to subset to those for whom we had greater confidence in an accurate diabetes classification helped to reduce disparities in health care use by observed diabetes status. In addition, while all correction methods greatly reduced the estimated association between these 2 chronic conditions, the DAG method had the largest impact on this estimate, suggesting use was a strong confounder of the association between these observed health outcomes. Since the presence of either chronic condition may cause increased health care use, it is also possible that controlling for this variable induced a small collider bias, producing an estimate that was lower than the sufficiency or IPW estimates. However, prior research suggests that the magnitude of this collider bias would be small relative to the confounding bias imparted when not controlling for this variable [14]. All corrected EHR estimates were still higher than the health survey point estimates, suggesting that the NYU patient population may not be generalizable, that there are residual biases in these estimates, or that there are other inherent differences between EHR and survey-based estimates. For example, individuals interacting with the NYU hospital system may be sicker and more likely to have multiple chronic conditions than those who receive care at independent primary care practices and conditioning on sicker patients could potentially introduce a collider bias if presence within the hospital system was a common effect of these conditions. This selection bias was not addressed in this work and is an important avenue for future research.

### Limitations

This study applied 2 bias correction frameworks to a large, diverse patient population and these findings can inform broader discussions on addressing misclassification of disease outcomes within epidemiologic studies using EHR data. However, there are limitations to these analyses. Importantly, internal or external validation samples were not available to inform computable phenotype sensitivity or specificity for these methods [8], and the sufficiency and IPW models relied on the strong assumption that sufficient cases had no misclassification in diabetes status. Sensitivity analyses were used to test this assumption, but it is possible that imposing sufficiency determinations generated a collider bias by selecting sicker individuals or those with diabetes [5], which could explain why these methods found higher odds of diabetes among those with asthma compared to the DAG method. That said, internal and external validation samples are often costly or time-intensive to obtain, so these methods offer an imperfect, yet feasible, solution within resource-constrained environments. In addition, methods focused on addressing differential misclassification of health outcomes, but there is potential for misclassification within other covariates. The hypothesized DAG likely represents a simplified depiction of information biases within these data. In particular, a large proportion of patients had an unknown race or ethnicity, and the BISG imputation methods used may have differential performance by race or ethnicity or marital status [35]. Overall, determining the accuracy of the EHR-based estimates was challenging due to the wide CIs for survey-based estimates, but EHR-based associations had greatly improved precision due to the diversity and larger sample size of these data.

Additionally, comparisons were made to estimates from 2 health surveys, which have distinct biases that were not addressed in this analysis. The BRFSS is limited to self-reported health outcomes, which can be prone to misclassification. However, evidence suggests that self-reported diabetes status may have good validity relative to other chronic conditions [4,36,37]. Physical measurements from NHANES may improve the classification of diabetes status among those who are unaware or undiagnosed [15]. However, the smaller sample size of this survey requires multiyear pooled analyses, which can be biased by changes in screening or diagnostic criteria over time. For example, in 2015, the American Diabetes Association lowered the recommended BMI screening threshold for Asian American individuals to better account for the differential risk of diabetes at equivalent BMI levels, which could change the burden of undetected diabetes within this subgroup across time [38]. The complementary strengths of these 2 data sources may help to remedy these unaddressed biases. However, differences in the targets for inference could have contributed to the observed differences between the EHR and survey estimates. Survey estimates reflect national data, which may not be transportable to this New York City patient population. Although local versions of these health surveys are available, sample sizes were too small to produce reliable associations. Covariate definitions also varied across data sources. Importantly, individual-level income was available within the survey data but was unavailable in the EHR data. The use of neighborhood-level poverty likely resulted in residual confounding in all EHR-based ORs for asthma, which may have contributed to the positive relative differences compared to survey-based estimates.

### Conclusions

EHRs offer a compelling data source for public health research; however, differential misclassification of disease status has the potential to bias the results of these studies. Methods to control for factors that affect misclassification using a causal framework, particularly when an informed presence bias can be hypothesized to strongly confound the exposure-outcome relationship, should be considered to help produce valid estimates of risk factor associations. The next steps include applying these methods to additional exposure-outcome relationships and incorporating the longitudinal nature of EHR data to assess causal relationships between chronic conditions.

## Supplementary material

10.2196/58085 Multimedia Appendix 1 Additional details on methods and supplementary tables and figures.

## Abbreviations

BISG: Bayesian Improved Surname Geocoding
BRFSS: Behavioral Risk Factor Surveillance System
DAG: directed acyclic graph
DM: diabetes
EHR: electronic health record
IPW: inverse probability weighting
NHANES: National Health and Nutrition Examination Survey
NYU: New York University
OR: odds ratio
