# Supplementary material for: Addressing Information Biases Within Electronic Health Record Data to Improve the Examination of Epidemiologic Associations With Diabetes Prevalence Among Young Adults: Cross-Sectional Study
Source: JMIR Med Inform. 2024 Oct 1;12:e58085. doi: 10.2196/58085 (PMC11460830; doi:10.2196/58085)
Supplement: Multimedia Appendix 1 [file medinform-v12-e58085-s001.docx]

**Additional Details on Methods**

**Naïve Model:**

The “naïve” model represented an approach where potential misclassification in diabetes status was not considered or addressed. Naïve models were estimated by fitting logistic regression models for observed diabetes status (DM*) on the full patient sample. ORs for race/ethnicity were adjusted for age and sex, and ORs for asthma were adjusted for age, sex, race/ethnicity, Medicaid insurance status, obesity, and neighborhood poverty level.

**Sufficiency Model:**

The “sufficiency” model represented an approach where misclassification in diabetes status was assumed to be akin to “missing completely at random” and could be addressed by restricting analyses to those with sufficient or complete data. Missingness was not directly observable since our definition for diabetes relied on evidence within the EHR (e.g., diagnosis codes, prescriptions for medications) to classify patients as having diabetes and assumed absence of diabetes without such evidence. For this reason, we defined data sufficiency criteria to create a subpopulation for which we had high confidence in the accuracy of their diabetes classification. Since diabetes is a rare disease among the young adult population, we assumed our algorithm had near-perfect specificity and misclassification largely occurred from missing data among individuals with diabetes who are falsely classified as non-diabetic (e.g., a patient had a diagnosis for diabetes at an external health system that was not present in the NYU Langone Health EHR).^1^ The subpopulation with sufficient data was defined to be those with at least one encounter with an endocrinology review of systems or those who were classified as diabetic. Sufficiency models were estimated by fitting a logistic regression model for DM* on the subset of patients who were hypothesized to have sufficient data. ORs for race/ethnicity were adjusted for age and sex, and ORs for asthma were adjusted for age, sex, race/ethnicity, Medicaid insurance status, obesity, and neighborhood poverty level.

**IPW Model:**

The “IPW” model represented an approach where misclassification in diabetes status was assumed to be akin to “missing at random” and could be addressed through inverse probability weighting (IPW) of the subpopulation with sufficient data. We hypothesized that missing health outcomes would be predicted by demographics (e.g., differential screening by race/ethnicity), healthcare utilization (e.g., informed presence bias), and neighborhood (e.g., degree of continuity of care within the health system by catchment area). We estimated the probability of having sufficient data, defined as those with at least one encounter with an endocrinology review of systems or those who were classified as diabetic, using a multilevel logistic regression model including all demographic (age, sex, race/ethnicity, Medicaid insurance status, and neighborhood poverty level) and healthcare utilization variables (total encounters, duration within the NYU Langone Health system, presence of at least one routine health exam, presence of at least one diabetes-related lab) and a random intercept for neighborhood defined by Public Use Microdata Areas. Stabilized IPW weights were then calculated as the inverse of the predicted probability of having sufficient data multiplied by the overall probability of having sufficient data. The models were estimated by fitting a logistic regression model for DM* on the subset of patients defined as having sufficient data, weighted for the stabilized IPW weights. ORs for race/ethnicity were adjusted for age and sex, and ORs for asthma were adjusted for age, sex, race/ethnicity, Medicaid insurance status, obesity, and neighborhood poverty level.

**DAG Model:**

The “DAG” model represented an approach where we constructed a directed acyclic graph (DAG) to help us identify and subsequently control for variables that could affect misclassification of disease status. In this scenario, we hypothesized that the number of healthcare encounters could affect misclassification of both asthma and diabetes through an informed presence bias and could also be an effect of these health outcomes, consistent with prior research (Appendix Figure 1).^2,3^ DAG models were estimated by fitting a logistic regression model for DM* using the full patient sample. ORs for race/ethnicity were adjusted for age, sex, and number of encounters, and ORs for asthma were adjusted for age, sex, race/ethnicity, Medicaid insurance status, obesity, neighborhood poverty level, and number of encounters.

**Table S1.** Case-insensitive search terms for endocrinology review of systems.

| **Endocrinology Key Terms** | **Review of System Key Terms** |
| --- | --- |
| endocrin | ros |
| endo: | review of system |
| endo- |  |

**Figure S1.** Hypothesized directed acyclic graph for information bias and associations between asthma and diabetes.


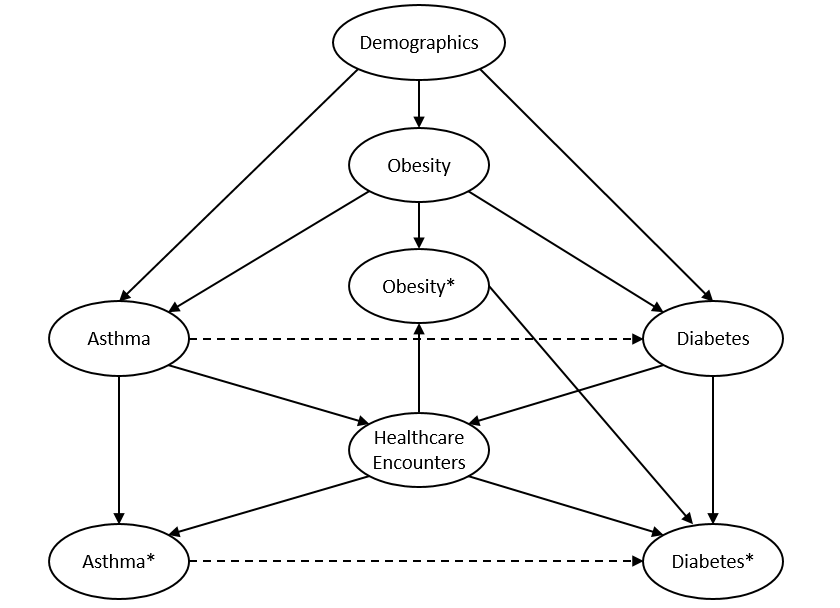


*Observed disease status

**Table S2.** Odds ratios for diabetes by race or ethnicity and asthma, health survey estimates.

|  | **BRFSS^a^** | **BRFSS – SA^b^** | **NHANES^c^** | **NHANES – SA^d^** |
| --- | --- | --- | --- | --- |
| **Race/Ethnicity**^e^  (Ref = White) |  |  |  |  |
| Black | 1.51 (1.3-1.75) | 1.49 (1.30-1.70) | 1.59 (1.18-2.14) | 1.32 (0.92-1.88) |
| Latino | 1.53 (1.32-1.77) | 1.51 (1.33-1.71) | 1.54 (1.14-2.07) | 1.41 (0.99-2.00) |
| Asian | 1.08 (0.81-1.45) | 1.18 (0.91-1.52) | 1.04 (0.71-1.52) | 0.68 (0.41-1.10) |
| Other | 1.62 (1.28-2.04) | 1.64 (1.34-2.00) | 1.47 (0.9-2.39) | 1.16 (0.68-2.00) |
| **Asthma**^f^  (Ref = No) | 1.23 (1.09-1.4) | 1.34 (1.17-1.53) | 1.38 (1.01-1.91) | 1.32 (0.90-1.93) |

*^a^ Associations with self-reported diabetes diagnosis observed among Behavioral Risk Factor Surveillance System Survey 2019 respondents aged 18-44 years who report having a personal healthcare provider.*

*^b^ BRFSS sensitivity analysis: associations with self-reported diabetes diagnosis observed among Behavioral Risk Factor Surveillance System Survey 2019 respondents aged 18-44 years.*

*^c^ Associations with self-reported diabetes diagnosis or undiagnosed diabetes (A1C≥6.5% or fasting glucose≥126mm/Hg) observed among National Health and Nutrition Examination Survey 2013-March 2020 respondents aged 18-44 years.*

*^d^ NHANES Sensitivity analysis: with self-reported diabetes diagnosis observed among National Health and Nutrition Examination Survey 2013-March 2020 respondents aged 18-44 years.*

*^e^ ORs for race/ethnicity estimated in reference to non-Hispanic White controlling for age and sex.*

*^f^ ORs for asthma estimated in reference to non-asthmatic controlling for age, sex, race/ethnicity, obesity, insurance status, and poverty level.*

**Table S3.** Odds ratios for diabetes by race or ethnicity and asthma, EHR-based estimates.

|  | **Naive^a^** | **Sufficient Case^b^** | **IPW^c^** | **Causal^d^** |
| --- | --- | --- | --- | --- |
| **Race/Ethnicity**^e^  (Ref = White) |  |  |  |  |
| Black | 1.79 (1.7-1.88) | 1.68 (1.59-1.76) | 1.73 (1.64-1.83) | 1.75 (1.67-1.84) |
| Latino | 1.93 (1.85-2.01) | 1.76 (1.69-1.84) | 1.75 (1.67-1.84) | 1.64 (1.57-1.71) |
| Asian | 1.11 (1.04-1.18) | 1.18 (1.11-1.26) | 1.26 (1.18-1.34) | 1.13 (1.06-1.2) |
| Other | 1.41 (1.3-1.51) | 1.46 (1.35-1.58) | 1.57 (1.45-1.7) | 1.4 (1.3-1.5) |
| **Asthma**^f^  (Ref = No) | 3.01 (2.86-3.18) | 1.87 (1.77-1.97) | 1.79 (1.67-1.92) | 1.42 (1.34-1.51) |

*^a^ Associations with EHR-defined diabetes status observed among NYC resident NYU patient population with an inpatient or outpatient encounter from 2017-2018.*

*^b^ Associations with EHR-defined diabetes status observed among NYC resident NYU patient population with an inpatient or outpatient encounter from 2017-2018 with sufficient records, as defined as those with a review of systems for endocrinology.*

*^c^ Associations with EHR-defined diabetes status observed among NYC resident NYU patient population with an inpatient or outpatient encounter from 2017-2018 with sufficient records, as defined as those with a review of systems for endocrinology, using IPW weighting the sufficient case subset with stabilized IPW.*

*^d^ Associations with EHR-defined diabetes status observed among NYC resident NYU patient population with an inpatient or outpatient encounter from 2017-2018, using the DAG controlling for total number of encounters.*

*^e^ ORs for race/ethnicity estimated in reference to non-Hispanic White controlling for age and sex.*

*^f^ ORs for asthma estimated in reference to non-asthmatic controlling for age, sex, race/ethnicity, obesity, insurance status, and poverty level.*

**Table S4.** Sensitivity analyses of sufficient case definitions: descriptive summary of NYU patient population by diabetes status.

|  | **≥ 1 Endocrinology Review of Systems** | | **≥ 1 Endocrinology & Respiratory Review of Systems** | | **≥ 1 DM-related Lab & BMI Measurement** | |
| --- | --- | --- | --- | --- | --- | --- |
|  | **Non-Diabetic** | **Diabetic** | **Non-Diabetic** | **Diabetic** | **Non-Diabetic** | **Diabetic** |
| **Total** | 166992 (94.5) | 9698 (5.5) | 289203 (95.9) | 12366 (4.1) | 190369 (94.1) | 11898 (5.9) |
| **Age** (30-44) | 104005 (62.3) | 7519 (77.5) | 177944 (61.5) | 9622 (77.8) | 122123 (64.2) | 9248 (77.7) |
| **Sex** (Male) | 65240 (39.1) | 4017 (41.4) | 112878 (39.0) | 5112 (41.3) | 71579 (37.6) | 4982 (41.9) |
| **Medicaid** | 37759 (22.6) | 2902 (29.9) | 64665 (22.4) | 3754 (30.4) | 46826 (24.6) | 3796 (31.9) |
| **Raw Race/ Ethnicity** | |  |  |  |  |  |
| White | 73996 (44.3) | 3412 (35.2) | 126615 (43.8) | 4452 (36.0) | 83192 (43.7) | 4248 (35.7) |
| Black | 18847 (11.3) | 1691 (17.4) | 32195 (11.1) | 2053 (16.6) | 21453 (11.3) | 1912 (16.1) |
| Latino | 27479 (16.5) | 2501 (25.8) | 46955 (16.2) | 3122 (25.2) | 35439 (18.6) | 3098 (26.0) |
| Asian/PI | 13227 (7.9) | 672 (6.9) | 22359 (7.7) | 888 (7.2) | 16727 (8.8) | 973 (8.2) |
| Other | 11709 (7.0) | 747 (7.7) | 20925 (7.2) | 980 (7.9) | 14891 (7.8) | 972 (8.2) |
| Missing | 21734 (13.0) | 675 (7.0) | 40154 (13.9) | 871 (7.0) | 18667 (9.8) | 695 (5.8) |
| **Imputed Race/ Ethnicity** | |  |  |  |  |  |
| White | 86629 (51.9) | 3744 (38.6) | 149693 (51.8) | 4872 (39.4) | 94025 (49.4) | 4603 (38.7) |
| Black | 21829 (13.1) | 1832 (18.9) | 37656 (13.0) | 2233 (18.1) | 24033 (12.6) | 2046 (17.2) |
| Latino | 33696 (20.2) | 2821 (29.1) | 58420 (20.2) | 3534 (28.6) | 41883 (22.0) | 3460 (29.1) |
| Asian/PI | 16599 (9.9) | 787 (8.1) | 28523 (9.9) | 1037 (8.4) | 19889 (10.4) | 1096 (9.2) |
| Other | 8239 (4.9) | 514 (5.3) | 14911 (5.2) | 690 (5.6) | 10539 (5.5) | 693 (5.8) |
| **Any BMI** | 163127 (97.7) | 9651 (99.5) | 281030 (97.2) | 12299 (99.5) | 190369 (100) | 11898 (100) |
| **Obese** | 34593 (20.7) | 4499 (46.4) | 58389 (20.2) | 5626 (45.5) | 42429 (22.3) | 5294 (44.5) |
| **Encounters*** | 22.27 (28.19) | 55.41 (60.49) | 18.59 (24.36) | 49.43 (56.13) | 23.09 (27.53) | 50.71 (56.68) |
| **Duration*** | 2.26 (1.97) | 3.09 (2.02) | 2.03 (1.95) | 2.96 (2.03) | 2.50 (1.98) | 3.05 (2.03) |
| **Routine Medical Exam** | 77927 (46.7) | 3927 (40.5) | 104336 (36.1) | 4456 (36.0) | 98508 (51.7) | 4421 (37.2) |
| **DM-related Lab**^a^ | 112752 (67.5) | 8790 (90.6) | 169914 (58.8) | 10933 (88.4) | 190369 (100) | 11898 (100) |
| **PUMA Coverage** |  |  |  |  |  |  |
| < 10 % | 28143 (16.9) | 2215 (22.8) | 49254 (17.0) | 2718 (22.0) | 30787 (16.2) | 2543 (21.4) |
| 10-<20% | 57185 (34.2) | 3244 (33.5) | 96460 (33.4) | 4074 (32.9) | 62652 (32.9) | 3813 (32.0) |
| 20-<30% | 57597 (34.5) | 2808 (29.0) | 101763 (35.2) | 3612 (29.2) | 66311 (34.8) | 3564 (30.0) |
| 30-<40% | 24067 (14.4) | 1431 (14.8) | 41726 (14.4) | 1962 (15.9) | 30619 (16.1) | 1978 (16.6) |
| **Asthma** (Yes) | 11172 (6.7) | 1563 (16.1) | 15887 (5.5) | 1819 (14.7) | 14039 (7.4) | 1854 (15.6) |

*^a^ Including all A1c, random blood glucose, and fasting blood glucose lab results.*

**Presented as mean (standard deviation).*

**Table S5.** Sensitivity analyses of sufficient case definitions: odds ratios for diabetes by race or ethnicity and asthma, EHR-based estimates sufficiency and IPW estimates.

|  | **≥ 1 Endocrinology Review of Systems** | | **≥ 1 Endocrinology & Respiratory Review of Systems** | | | **≥ 1 DM-related Lab & BMI Measurement** | | |
| --- | --- | --- | --- | --- | --- | --- | --- | --- |
|  | **Sufficiency^a^** | **IPW^b^** | | **Sufficiency^a^** | **IPW^b^** | | **Sufficiency ^a^** | **IPW^b^** |
| **Race/Ethnicity**^c^  (Ref = White) |  |  | |  |  | |  |  |
| Black | 1.89 (1.78-2.01) | 1.72 (1.64-1.81) | | 1.88 (1.78-2) | 2.03 (1.89-2.18) | | 1.72 (1.63-1.82) | 1.76 (1.66-1.87) |
| Latino | 1.97 (1.87-2.08) | 1.74 (1.67-1.82) | | 1.95 (1.86-2.06) | 2.07(1.94-2.2) | | 1.73 (1.65-1.81) | 1.74 (1.65-1.83) |
| Asian | 1.11 (1.02-1.2) | 0.97 (0.91-1.04) | | 1.09 (1.01-1.19) | 1.13(1.03-1.24) | | 1.14 (1.06-1.22) | 1.3 (1.21-1.39) |
| Other | 1.38 (1.25-1.52) | 1.24 (1.14-1.35) | | 1.36 (1.24-1.5) | 1.45(1.3-1.62) | | 1.31 (1.2-1.42) | 1.42 (1.3-1.55) |
| **Asthma**^d^  (Ref = No) | 1.83 (1.71-1.96) | 1.73 (1.66-1.81) | | 2.23 (2.1-2.37) | 2.28 (2.1-2.46) | | 2.06 (1.95-2.17) | 1.98 (1.85-2.12) |

*^a^ Associations with EHR-defined diabetes status observed among NYC resident NYU patient population with an inpatient or outpatient encounter from 2017-2018 with sufficient records, as defined by column header.*

*^b^ Associations with EHR-defined diabetes status observed among NYC resident NYU patient population with an inpatient or outpatient encounter from 2017-2018 with sufficient records, as defined by column header.*

*^c^ ORs for race/ethnicity estimated in reference to non-Hispanic White controlling for age and sex.*

*^d^ ORs for asthma estimated in reference to non-asthmatic controlling for age, sex, race/ethnicity, obesity, Medicaid insurance status, and poverty level.*

**Table S6.** Descriptive summary of NYU patient population by data sufficiency status with standardized mean differences.^a-b^

|  | Total Sample | Non-Sufficient Case | Sufficient Case^a^ | Standardized Mean Difference |
| --- | --- | --- | --- | --- |
|  | n(%) | n(%) | n(%) |  |
|  |  |  |  |  |
| **Total** | 454,612 (100) | 273,576 (60.4) | 181,036 (39.8) |  |
| **Age (mean (sd))** | 32.13 (7.11) | 32.02 (7.13) | 32.31 (7.07) | 0.041 |
| **Sex (Male)** | 171968 (37.8) | 100964 (36.9) | 71004 (39.2) | 0.048 |
| **Medicaid Insurance (Yes)** | 100979 (22.2) | 59001 (21.6) | 41978 (23.2) | 0.039 |
| **Raw Race/Ethnicity** |  |  |  | 0.317 |
| White | 190225 (41.8) | 111123 (40.6) | 79102 (43.7) |  |
| Black | 45509 (10.0) | 24442 (8.9) | 21067 (11.6) |  |
| Latino | 62989 (13.9) | 32157 (11.8) | 30832 (17.0) |  |
| Asian/PI | 35262 (7.8) | 20947 (7.7) | 14315 (7.9) |  |
| Other | 32525 (7.2) | 19669 (7.2) | 12856 (7.1) |  |
| Missing | 88102 (19.4) | 65238 (23.8) | 22864 (12.6) |  |
| **Imputed Race/Ethnicity** |  |  |  | 0.092 |
| White | 237057 (52.1) | 144783 (52.9) | 92274 (51.0) |  |
| Black | 57709 (12.7) | 33439 (12.2) | 24270 (13.4) |  |
| Latino | 86679 (19.1) | 49131 (18.0) | 37548 (20.7) |  |
| Asian/PI | 49170 (10.8) | 31288 (11.4) | 17882 (9.9) |  |
| Other | 23997 (5.3) | 14935 (5.5) | 9062 (5.0) |  |
| **Any Recorded BMI (Yes)** | 367903 (80.9) | 190916 (69.8) | 176987 (97.8) | 0.820 |
| **Encounters^c^ (mean (sd))** | 15.23 (23.51) | 9.41 (13.15) | 24.03 (31.60) | 0.604 |
| **Duration^d^ (mean (sd))** | 1.84 (1.93) | 1.53 (1.83) | 2.31 (1.98) | 0.405 |
| **≥ 1 Routine Medical Exam (Yes)** | 115249 (25.4) | 32786 (12.0) | 82463 (45.6) | 0.798 |
| **≥ 1 DM-related Lab (Yes)^b^** | 205408 (45.2) | 80728 (29.5) | 124680 (68.9) | 0.857 |
| **Neighborhood Coverage^e^** |  |  |  | 0.090 |
| < 10 % | 79563 (17.5) | 48320 (17.7) | 31243 (17.3) |  |
| 10-<20% | 143907 (31.7) | 82148 (30.0) | 61759 (34.1) |  |
| 20-<30% | 163076 (35.9) | 101293 (37.0) | 61783 (34.1) |  |
| 30-<40% | 68066 (15.0) | 41815 (15.3) | 26251 (14.5) |  |
| **Asthma (Yes)** | 19240 (4.2) | 6167 (2.3) | 13073 (7.2) | 0.235 |
| **Obese (Yes)** | 79580 (17.5) | 38819 (14.2) | 40761 (22.5) | 0.216 |

*^a^ Sufficient cases defined as those with at least one encounter with an endocrinology review of systems or those who were classified as diabetic through the computable phenotype of having at least two encounter diagnoses for diabetes, one encounter diagnosis and at least two elevated A1C lab results ≥6.5%, or at least one anti-diabetes prescription medication.*

*^b^ Including all A1c, random blood glucose, and fasting blood glucose lab results.*

*^c^ Number of encounters.*

*^d^ Number of years in the health system.*

*^e^ Proportion of individuals residing in the Public Use Microdata Area (PUMA) neighborhood who are present within the EHR system.*

**Table S7.** Descriptive summary of NYU patient population by diabetes status with standardized mean differences.^a-b^

|  | Naive  Diabetes Status | |  | Sufficient  Diabetes Status^a^ | |  |
| --- | --- | --- | --- | --- | --- | --- |
|  | Non-Diabetic | Diabetic | Standardized Mean Difference | Non-Diabetic | Diabetic | Standardized Mean Difference |
|  | n(%) | n(%) |  | n(%) | n(%) |  |
|  |  |  |  |  |  |  |
| **Total** | 440568 (96.9) | 14044 (3.1) |  | 166992 (92.2) | 14044 (7.8) |  |
| **Age (30-44 years)** | 273216 (62.0) | 10843 (77.2) | 0.335 | 104005 (62.3) | 10843 (77.2) | 0.329 |
| **Sex (Male)** | 166204 (37.7) | 5764 (41.0) | 0.068 | 65240 (39.1) | 5764 (41.0) | 0.040 |
| **Medicaid Insurance (Yes)** | 96760 (22.0) | 4219 (30.0) | 0.185 | 37759 (22.6) | 4219 (30.0) | 0.169 |
| **Raw Race/Ethnicity** |  |  | 0.450 |  |  | 0.292 |
| White | 185119 (42.0) | 5106 (36.4) |  | 73996 (44.3) | 5106 (36.4) |  |
| Black | 43289 (9.8) | 2220 (15.8) |  | 18847 (11.3) | 2220 (15.8) |  |
| Latino | 59636 (13.5) | 3353 (23.9) |  | 27479 (16.5) | 3353 (23.9) |  |
| Asian/PI | 34174 (7.8) | 1088 (7.7) |  | 13227 (7.9) | 1088 (7.7) |  |
| Other | 31378 (7.1) | 1147 (8.2) |  | 11709 (7.0) | 1147 (8.2) |  |
| Missing | 86972 (19.7) | 1130 (8.0) |  | 21734 (13.0) | 1130 (8.0) |  |
| **Imputed Race/Ethnicity** |  |  | 0.293 |  |  | 0.259 |
| White | 231412 (52.5) | 5645 (40.2) |  | 86629 (51.9) | 5645 (40.2) |  |
| Black | 55268 (12.5) | 2441 (17.4) |  | 21829 (13.1) | 2441 (17.4) |  |
| Latino | 82827 (18.8) | 3852 (27.4) |  | 33696 (20.2) | 3852 (27.4) |  |
| Asian/PI | 47887 (10.9) | 1283 (9.1) |  | 16599 (9.9) | 1283 (9.1) |  |
| Other | 23174 (5.3) | 823 (5.9) |  | 8239 (4.9) | 823 (5.9) |  |
| **Any Recorded BMI (Yes)** | 354043 (80.4) | 13860 (98.7) | 0.627 | 163127 (97.7) | 13860 (98.7) | 0.075 |
| **Obese (Yes)** | 73412 (16.7) | 6168 (43.9) | 0.621 | 34593 (20.7) | 6168 (43.9) | 0.512 |
| **Encounters^c^ (mean (sd))** | 14.29 (21.16) | 44.90 (54.27) | 0.743 | 22.27 (28.19) | 44.90 (54.27) | 0.523 |
| **Duration^d^ (mean (sd))** | 1.81 (1.92) | 2.87 (2.06) | 0.532 | 2.26 (1.97) | 2.87 (2.06) | 0.301 |
| **≥1 Routine Medical Exam (Yes)** | 110713 (25.1) | 4536 (32.3) | 0.159 | 77927 (46.7) | 4536 (32.3) | 0.297 |
| **≥ 1 DM-related Lab (Yes)^b^** | 193480 (43.9) | 11928 (84.9) | 0.948 | 112752 (67.5) | 11928 (84.9) | 0.418 |
| **Neighborhood Coverage^e^** |  |  | 0.154 |  |  | 0.151 |
| < 10 % | 76463 (17.4) | 3100 (22.1) |  | 28143 (16.9) | 3100 (22.1) |  |
| 10-<20% | 139333 (31.6) | 4574 (32.6) |  | 57185 (34.2) | 4574 (32.6) |  |
| 20-<30% | 158890 (36.1) | 4186 (29.8) |  | 57597 (34.5) | 4186 (29.8) |  |
| 30-<40% | 65882 (15.0) | 2184 (15.6) |  | 24067 (14.4) | 2184 (15.6) |  |
| **Asthma (Yes)** | 17339 (3.9) | 1901 (13.5) | 0.345 | 11172 (6.7) | 1901 (13.5) | 0.229 |

*^a^ Sufficient cases defined as those with at least one encounter with an endocrinology review of systems or those who were classified as diabetic through the computable phenotype of having at least two encounter diagnoses for diabetes, one encounter diagnosis and at least two elevated A1C lab results ≥6.5%, or at least one anti-diabetes prescription medication.*

*^b^ Including all A1c, random blood glucose, and fasting blood glucose lab results.*

*^c^ Number of encounters.*

*^d^ Number of years in the health system.*

*^e^ Proportion of individuals residing in the Public Use Microdata Area (PUMA) neighborhood who are present within the EHR system.*

**References**

1. Quan H, Li B, Saunders LD, et al. Assessing validity of ICD-9-CM and ICD-10 administrative data in recording clinical conditions in a unique dually coded database. *Health services research.* 2008;43(4):1424-1441.

2. Goldstein BA, Bhavsar NA, Phelan M, Pencina MJ. Controlling for Informed Presence Bias Due to the Number of Health Encounters in an Electronic Health Record. *Am J Epidemiol.* 2016;184(11):847-855.

3. Phelan M, Bhavsar NA, Goldstein BA. Illustrating Informed Presence Bias in Electronic Health Records Data: How Patient Interactions with a Health System Can Impact Inference. *EGEMS (Wash DC).* 2017;5(1):22-22.
